# Impairment of Rat Fetal Beta-Cell Development by Maternal Exposure to Dexamethasone during Different Time-Windows

**DOI:** 10.1371/journal.pone.0025576

**Published:** 2011-10-03

**Authors:** Olivier Dumortier, Nicolas Theys, Marie-Thérèse Ahn, Claude Remacle, Brigitte Reusens

**Affiliations:** Laboratoire de Biologie Cellulaire, Université catholique de Louvain, Institut des Sciences de la Vie, Louvain-la-Neuve, Belgium; Flinders University, Australia

## Abstract

**Aim:**

Glucocorticoids (GCs) take part in the direct control of cell lineage during the late phase of pancreas development when endocrine and exocrine cell differentiation occurs. However, other tissues such as the vasculature exert a critical role before that phase. This study aims to investigate the consequences of overexposure to exogenous glucocorticoids during different time-windows of gestation for the development of the fetal endocrine pancreas.

**Methods:**

Pregnant Wistar rats received dexamethasone acetate in their drinking water (1 µg/ml) during the last week or throughout gestation. Fetuses and their pancreases were analyzed at day 15 and 21 of gestation. Morphometrical analysis was performed on pancreatic sections after immunohistochemistry techniques and insulin secretion was evaluated on fetal islets collected in vitro.

**Results:**

Dexamethasone given the last week or throughout gestation reduced the beta-cell mass in 21-day-old fetuses by respectively 18% or 62%. This was accompanied by a defect in insulin secretion. The alpha-cell mass was reduced similarly. Neither islet vascularization nor beta-cell proliferation was affected when dexamethasone was administered during the last week, which was however the case when given throughout gestation. When given from the beginning of gestation, dexamethasone reduced the number of cells expressing the early marker of endocrine lineage neurogenin-3 when analyzed at 15 days of fetal age.

**Conclusions:**

GCs reduce the beta- and alpha-cell mass by different mechanisms according to the stage of development during which the treatment was applied. In fetuses exposed to glucocorticoids the last week of gestation only, beta-cell mass is reduced due to impairment of beta-cell commitment, whereas in fetuses exposed throughout gestation, islet vascularization and lower beta-cell proliferation are involved as well, amplifying the reduction of the endocrine mass.

## Introduction

Epidemiological studies performed in distinct populations throughout the world have clearly reported that individuals who had a low birth weight or were thin at birth feature a higher prevalence of glucose intolerance and type 2 diabetes (T2D) during adult life [1], [2], [3], [4]. Indeed various nutritional changes during embryonic development have the potential of altering gene expression and modifying T2D susceptibility. Thus, a key question arises concerning the nature of the components of the intrauterine metabolic environment which initiate the deleterious cascade. Evidence supports elevated levels of maternal (and thus fetal) glucocorticoids may be a key unifying mechanism linking maternal malnutrition to increased diabetes risk in adult life [5], [6], [7]. Maternal use of glucocorticoids during pregnancy in human and experimental animal may cause low birth weight and intrauterine growth retardation (IUGR) and later may lead to chronic diseases such as hypertension and type 2 diabetes [8], [9], [10], [11]. In the pathogenesis of T2D, insulin resistance coupled to beta cell failure leads to chronic hyperglycemia defining diabetes. In growth-retarded animals or humans, functional disruption in multiple tissues including muscle, adipose tissue, liver, and pancreas is observed in adults. In such low birth weight offspring, one may wonder whether the beta cell alteration observed later in life has been already acquired *in utero* or is a consequence of an adaption to the disturbed insulin sensitivity of target tissues.

Shen et al (2003) have investigated the direct effect of a glucocorticoid excess during the last week of gestation in rat on the development of the endocrine pancreas in the progeny. Like others, they found that glucocorticoids treatment led to IUGR and showed that at 3 weeks of age, the IUGR progeny featured a normal distribution of beta and alpha cell within islets, but reported a lower level of insulin expression in beta cells compared to normal pancreas [12]. However Shen et al did not investigate the beta cell mass and function immediately after the treatment i.e.at the end of gestation or at birth.

In order to understand the mechanisms by which glucocorticoids may affect the pancreas development, Shen et al 2003 investigated also the role of glucocorticoids on pancreatic buds from mouse embryo *in vitro*. They used pancreatic buds from E11.5 mouse embryo and treated them with dexamethasone. They showed that the expression of Pancreatic and duodenal specific transcription factor (Pdx-1), a homeodomain protein that is expressed in the entire pancreatic anlagen at early stages, that declines during later embryonic stages in most of the pancreas but remains only in beta cells later in life, was not affected in an early stage of development but rather suppressed in a later phase, leading to less differentiated beta cells after one week of culture [12].

One year later, Gesina *et al.* (2004) further analyzed the role of glucocorticoids in the development of the endocrine pancreas. When pancreatic bud from normal E15.5 mouse embryo were cultured in presence of glucocorticoids, the number of endocrine precursor cells was not affected but fewer differentiated beta-cells and more differentiated acinar cells were reported [13]. They concluded that glucocorticoids favor exocrine pancreas development at the detriment of the endocrine tissue. Using transgenic mouse lacking glucocorticoid receptor (GR) they showed that the selective inactivation of the GR gene in insulin-expressing beta-cells in mice had no consequences on beta- or alpha-cell mass, whereas the absence of GR in the expression domain of Pdx-1 led to a twofold increased beta-cell mass, with increased islet numbers and size. In addition, they showed that GR does not appear necessary for early phases but its accurate dosage is required to unable beta cell mass expansion in later stages [14]. Since GR is very weakly expressed in the rodent pancreas before E14 [15], the organ should be rather insensitive to a direct action of glucocorticoids during the first two weeks of gestation. However, during this earlier period the proper development of the endocrine pancreas requires the specific influence of tissues like vasculature [16], [17].

Therefore, the aim of our study was to investigate the beta cell mass expansion and function at the end of gestation when glucocorticoids were given from the beginning of gestation and to compare the effects when given only during the last week. We demonstrate that in addition to compromising the beta and alpha cell differentiation, glucocorticoids given from the beginning of gestation inhibit the beta-cell proliferation as well as function and reduce the islet vascularization which presumably may contribute to amplify the reduction of the endocrine mass.

## Materials and Methods

### Ethics Statement

All procedures were performed with the approval of the Animal Ethics Committee of the Université catholique de Louvain (Permit number LA 1220028).

### Animals and prenatal treatments

Nulliparous 200 to 250 g female Wistar rats (Janvier, Le Genest Saint Isle, France) were mated with males overnight and copulation was verified next morning by inspection of vaginal smears. Midnight was arbitrarily considered as time of mating at day 0 of gestation. The pregnant females were individually housed at 25°C with 14 h light∶ 10 h dark cycle and had free access to water and food (20% protein wt/wt; Hope Farm, Woerden, the Netherlands). Dexamethasone acetate (1 µg/ml, dexamethasone 21-acetate; Sigma-Aldrich, St Louis, MO, USA) was given in the drinking water of the mother as previously described [18]. This doses is comparable to that given IP by Shen et al, 2003 in pregnant rats [12] and is of the same range as that given to pregnant women at risk of preterm delivery [19]. Three groups of animals were created. The DEX group received dexamethasone acetate in the drinking water (1 µg/mL) throughout gestation. The DEXL group received dexamethasone during the last week of gestation whereas the C group received normal drinking water. A minimum of 3 litters per group and per age and 3 fetuses per mother were analyzed in each experiment.

### Blood and tissue sampling and analysis

At day 20 of gestation, 24 hours before sacrifice, the dams were injected intraperitoneally with BrdU (50 mg/kg; Sigma-Aldrich). At day 21 or at day 15, the mothers were sacrificed and fetuses were rapidly removed. The fetal blood samples were collected at day 21 from the axillary vein, when the feto-maternal circulation was maintained. Fetuses were weighed and their pancreas, liver, placenta, adrenal glands and brain were removed and weighed. Pancreases from 3 fetuses per litter were used for immunohistochemistry, 3 others were used for the determination of islet vascularization and 3 others for the analysis of pancreatic insulin content.

For measurement of glucose concentration, 50 µL of blood was added to 500 µL HC104 (0.33 N) to precipitate proteins. After centrifugation, supernatants were kept at −20°C until analysis. Blood glucose was measured by the glucose oxidase method using glucose Trinder's reagent (Sigma, Saint-Louis, MO, USA and Stanbio Laboratory, Boerne, TX, USA). Plasma was prepared and kept at −20°C for the determination of insulin. Plasma insulin levels were assessed using the ultrasensitive rat insulin ELISA and the pancreatic insulin content using the high range rat insulin ELISA (Mercodia, Uppsala, Sweden).

### Fixation and tissue processing for immunohistochemistry

Pancreases from 15 and 21 day-old fetuses were fixed in 3.7% formalin solution, dehydrated, and embedded in paraffin. Tissue sections (7 µm) were collected on poly-L-lysine-coated glass slides. The slides were left at 37°C overnight and stored at 4°C until processed for immunohistochemistry.

### Immunohistochemistry

Tissue sections were submitted to a 10-min microwave treatment in a citrate buffer (Antigen Retrieval Citra Solution; Biogenex, Alphelys, Plaisir, France), permeabilized for 20 min with 0.1% Triton X-100 in Tris-buffered saline, and incubated 30 min with a blocking buffer solution (0.1% Tween 20/3% BSA in Tris-buffered saline) before a 4°C overnight incubation with the primary antibodies. Secondary antibodies were incubated for 1 h at room temperature. Primary antibodies were rabbit anti-PDX1 (1/1000) and rabbit anti- Neurogenin 3 (Ngn3) (NEUROG3) (1/2000) (generous gifts from Dr R. Scharfmann), rabbit anti-Pancreas specific transcription factor (PTF1A) (1/2000) (10), mouse anti-insulin (1/1000; Sigma), anti-glucagon (1/1000; Novo Nordisk, Bogsvaerd, Denmark ) and mouse anti-BrdU (1/200; Amersham Pharmacia Biotech Europe, Saclay, France), Secondary antibodies were anti-mouse AP Conjugate (1/200; Promega, Madisson, WI, USA), biotin conjugated anti-rabbit (1/200; Jackson Immuno Research Laboratories, West Grove, PA, USA), peroxidase-conjugated anti-rabbit (1/200; Promega). Biotin-coupled antibodies were revealed using peroxidase-conjugated streptavidin (Amersham Pharmacia Biotech). Peroxidase was detected with diaminobenzidine (Dako, Carpinteria, CA) and alkaline phosphatase with the Vector blue alkaline phosphatase substrate kit III (Vector Laboratories, Inc., Burlingam, USA).

### TUNEL assay

Assessment of cell death by apoptosis was examined on tissue section using the TUNEL method with an in situ cell death detection kit (Roche, Indianapolis, IN, USA). Total nuclei were stained in blue with DAPI. In addition to staining for apoptosis, insulin detection was performed as described above.

### Morphometrical measurements

In F15 fetuses, cells expressing NEUROG3 were counted on 3 pancreatic sections per animal, a total of six animals from 3 different mothers being analyzed per group. Pancreatic area was determined by computer-assisted measurements on the adjacent section stained with toluidine blue using an Axioskop2 Mot Plus Zeiss microscope coupled with the Zeiss KS 400 3.0 software (Carl Zeiss GmbH, Jena, Germany).

On pancreatic sections of 21 day-old fetuses, cells co-expressing PDX-1 and insulin as well as cells positive for BrdU and insulin were counted. Insulin and glucagon positive area was morphometrically measured on 3 sections per animal, a total of nine animals being analyzed per group. The beta-cell and alpha-cell fractions (%) were measured as the ratio of the insulin or glucagon positive cell area to the total tissue area on the entire section. The beta-cell and the alpha-cell mass was obtained by multiplying the beta-cell or the alpha-cell fraction by the weight of the pancreas, as previously described [20].

The profile diameter of the islets was calculated using the Zeiss KS 400 3.0 software. The diameter of islets was calculated assuming that the islets are spheroid structures. The Zeiss KS 400 3.0 software was calibrated to measure such as an islet a cluster of minimum 3 insulin positives cells.

### Tissue preparation and epon-embedding for vascularization measurements

Pancreases of 21-day old fetuses were placed in ice-cold fixative (2.5% v/v) glutaraldhehyde in 0.1 M phosphate buffer (pH 7.2) during 2 hours, rinsed and post-fixed in 1% osmium tetroxide in phosphate buffer for 1 hour. The pieces were washed in phosphate buffer, ethanol-dehydrated, and epon-embedded as described previously [20].

### Vascularization analysis

Vascularization was measured on semi-thin sections allowing visualizing the lumen of blood vessel (see results). Three semi-thin sections (1 µm) of epon-embedded pieces were randomly cut in each pancreas, and stained with toluidine blue. The area of the each islet presents on the 3 sections and of the area of the different blood vessels in each islet were measured, using NIH-Image 1.56 software in a Reichert Polyvar microscope (Wien, Austria). Volume density of blood vessels in the islet was calculated as follow: Total area of blood vessel/islet reported to the islet area. To estimate the number density of islet blood vessels, the number of capillaries was counted in each islet and reported to the islet area. Nine fetuses from 3 different litters were used in each group (C, DEXL, and DEX).

### Culture of fetal islets

Fetal neoformed islets were obtained as previously described [21] .Briefly, pancreases of 21 day-old fetuses were removed aseptically. All the preparation, including the pancreatic tissue digestion, was carried out with RPMI 1640 medium containing 11.1 mM glucose (Gibco, Grand Island, NY, U.S.A.). The medium was supplemented with 10% (v/v) heat-inactivated fetal bovine serum (Gibco) and antibiotics (penicillin 200 U/ml, streptomycin 0.2 mg/ml, Gibco). The pancreases were minced and digested with collagenase (Sigma-Aldrich, specific activity 381 U/ml, 1.6 mg/ml per 12 pancreases) at 37°C. The digestion was stopped by adding ice-cold medium. After washing, tissue samples were suspended in 10 ml medium and gently stirred at room temperature for 60 min. The digested pancreases were pelleted by centrifugation, and re-suspended in a ratio of one pancreas per ml of medium. Finally 2 ml of this suspension were poured into 35 mm Petri dishes (Falcon 3001; Falcon Plastics, Los Angeles, CA, USA). The culture dishes were incubated for 7 days at 37°C, in a humidified atmosphere of 5% CO_2_ in air. The culture medium was changed daily after the second day. During the culture, exocrine cells disappear rapidly, whereas fibroblasts and endocrine cells proliferate. The endocrine cells are first arranged in monolayers but progressively reorganize in islets essentially composed of beta-cells (90–95% of beta-cells) that aggregated progressively on the layer of non-endocrine cells and which gradually acquire the capacity to secrete insulin in response to glucose [22].

### Insulin secretion assay

All these experiments were performed using a Krebs-Ringer solution containing (mmol/L): NaCl, 120; KCl, 5; CaCl2, 2; MgCl2, 1; NaHCO3 which was supplemented with 5 g/L bovine serum albumin (BSA) (Fraction V, Calbiochem-Behring, San Diego, CA, USA). This solution was gassed with 95% O2/5% CO2 to maintain a pH of 7.4. Glucose was added into the incubation medium without correction for osmolarity. Batches of 10 free-floating fetal neoformed islets were picked up and incubated at 37°C in 1 mL of Krebs-Ringer medium containing glucose at 2.5 mmol/L or 16.7 mmol/L. After 120 min, the incubation medium was removed and placed in a watch glass to verify that no islet had been taken. Then, the medium was frozen until the insulin assay was performed. To determine insulin content, islets were collected under microscopic observation and homogenized by sonification (30 s, 40 W) in 0•5 ml acid-ethanol [0.15 mol/L HCl in 75% (v/v) ethanol in water] to extract insulin. To eliminate variations due to differences in individual batches of islets, insulin secretion during incubation was expressed as a percentage of the islet insulin content at the start of the incubation, which is referred to as fractional insulin release. The latter was obtained by adding the content measured at the end to the amount of released insulin. Islets size was determined by measuring the diameters of islets collected in vitro.

### Statistical analysis

All results were expressed as means ± SEM. The statistical significance of variations was evaluated with Prism software (GraphPad software INC., San Diego, CA, USA). Cell number, cell proliferation, beta-cell fraction and mass, blood vessel number and density were tested by a one way ANOVA followed by Newman-Keuls. P values<0.05 were considered significant.

## Results

### Maternal body weight gain and food intake during pregnancy

**Figure 1:**
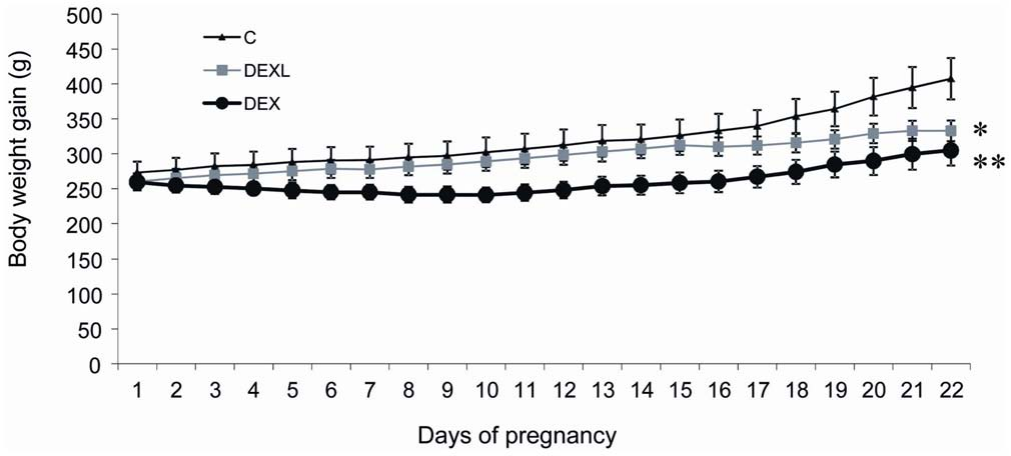
Maternal body weight during pregnancy. Dexamethasone was administered to the mother during the last week of gestation (DEXL) or throughout gestation (DEX). Values are means ± SEM, n = 4, **P<0.01, *P<0.05 vs Controls (C).

Dexamethasone impaired the normal body weight gain in pregnant animals in relation to the duration of the treatment (Figure 1). At day 21, the maternal weight was respectively reduced by 18.5% in the DEXL and by 25% in DEX groups. When the entire gestation period was taken into account, no significant difference in food intake between groups was apparent, but a small reduction was noted during 2–3 days after the first administration of dexamethasone (data not shown).

### Effect of dexamethasone on body and organ weight in fetuses

Litter size was similar in the three groups (range: 10–12 fetuses). Compared to Controls, fetal body weight was reduced by 18% when dexamethasone was administered during the last week of gestation and by 30% when given throughout gestation (Table 1). The weight of the pancreas, liver, adrenal gland, and placenta was reduced according to the time window of dexamethasone treatment (Table 1). The relative brain weight was significantly increased in dexamethasone treated fetuses suggesting that brain development was spared in these animals.

**Table 1:** Body (g) and organ weight (mg) of 21-day-old fetuses from control mother and mother treated with dexamethasone.

	C	DEXL	DEX
Fetus (n = 45)	5.47±0.06	4.49±0.06***	3.78±0.07*** $$$
Pancreas (n = 35)	31.4±0.74	27.1±0.62**	22.9±0.85*** $$
Relative pancreatic weight, mg/g body wt	5.72±0.08	6.06±0.12	6.04±0.16
Liver (n = 12)	316±11	211±8***	143±13*** $$$
Relative liver weight, mg/g body wt	57.2±1.8	50.2±1.7$$$	37.8±2.8***
Brain (n = 12)	181±4	179±3	177±6
Relative brain weight, mg/g body wt	32.5±0.6	41.5±1***	47.1±1.2*** $$
Adrenal (n = 20)	3.04±0.13	1.28±0.06***	1.24±0.05***
Relative adrenal weight, mg/g body wt	0.55±0.02	0.29±0.01***	0.33±0.02***
Placenta (n = 45)	632±20	454±11***	350±10*** $$$
Relative placenta weight, mg/g body wt	115±3	102±3**	93±25***

Values are means ± SEM,

***P<0.001,

**P<0.01, vs C ;

$$$ *P*<0.001,

$$ *P*<0.01, vs DEXL.

### Dexamethasone and fetal pancreas development

**Figure 2:**
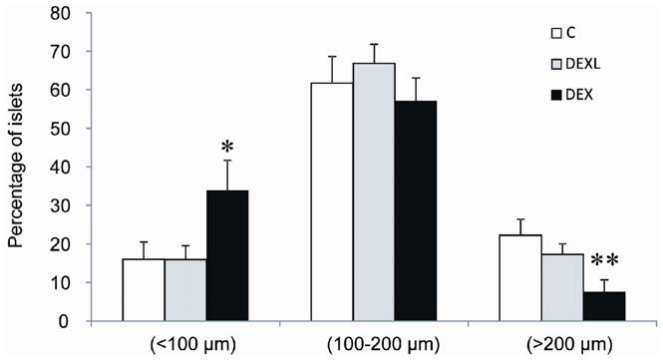
Islet size distribution in the 21-day-old fetuses. Effect of dexamethasone on size-frequency distribution of the 21-day-old rat pancreatic islets. Values are means ± SEM, n = 9, * P<0.05, ** P<0.01, vs C.

No disturbance of the intra-islet organization of the different endocrine cell populations was observed in DEXL and DEX groups at the end of gestation. Indeed, beta-cells were located in the core of islets and were surrounded by glucagon and somatostatin cells (not shown). Morphometrical analysis revealed however that the beta- and the-alpha mass was significantly reduced in the DEX and DEXL groups (Table 2), and the inhibitory effect of dexamethasone was significantly more marked when given from the first day of gestation than during the last week only. Dexamethasone given from the first day of gestation significantly increased the number of small islets and reduced the number of large islets compared to control (Figure 2).

**Table 2:** Beta- and Alpha-cell mass of 21-day-old fetuses from control mother and mother treated with dexamethasone.

	C	DEXL	DEX
Beta-cell mass (mg)	1.75±0.2	1.38±0.07*	0.75±0.1** $$
Relative beta-cell mass mg/g body wt	0.32±0.04	0.31±0.05	0.20±0.03**
Alpha-cell mass (mg)	0.51±0.08	0.28±0.05**	0.18±0.02***
Relative alpha-cell mass mg/g body wt	0.09±0.006	0.06±0.006*	0.04±0.007**

Values are means ± SEM, n = 9,

*P<0.05,

**P<0.01,

***P<0.001 vs C;

$$ P<0.01, vs DEXL.

### Effects of dexamethasone on beta-cell proliferation, apoptosis and pancreatic transcription factors

**Figure 3:**
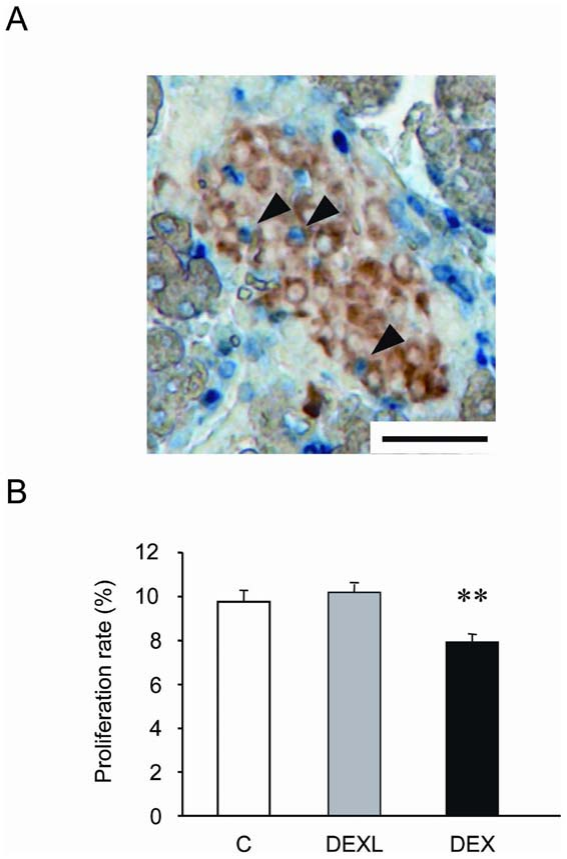
Effect of dexamethasone on BrdU incorporation in beta-cells of 21-day-old fetuses. (A) Beta-cells in S phase (blue) were counted on pancreatic section in colocalisation with insulin (brown). Beta-cells that are proliferating are indicated with arrowheads. (B) Percentage of proliferating beta-cells over total beta-cells. A total of 4500 to 6000 nuclei were counted in each group. Values are means ± SEM, n = 9 **P<0.01 vs Controls. Scale bar = 50 µm.

Proliferation, apoptosis and neogenesis are cellular mechanisms potentially involved in the modulation of the beta-cell mass. BrdU incorporation into DNA was used as an index of cell proliferation (Figure 3A). The percentage of beta-cells positive for BrdU was similar in Controls and in fetuses overexposed to GCs during the last week of gestation (Figure 3B), but when dexamethasone was administered throughout gestation, the beta-cell proliferation was reduced by 20% (Figure 3B).

Apoptosis was revealed by DNA fragmentation assessed by molecular cytochemistry by counting the TUNEL positive nuclei in insulin-positive cells on the last day of gestation. In every groups, the beta-cell apoptotic rate was very low (range: 0.1%–0.2%, data not shown) suggesting that apoptosis was not implicated in the impairment of islet development by dexamethasone at this age.

**Figure 4:**
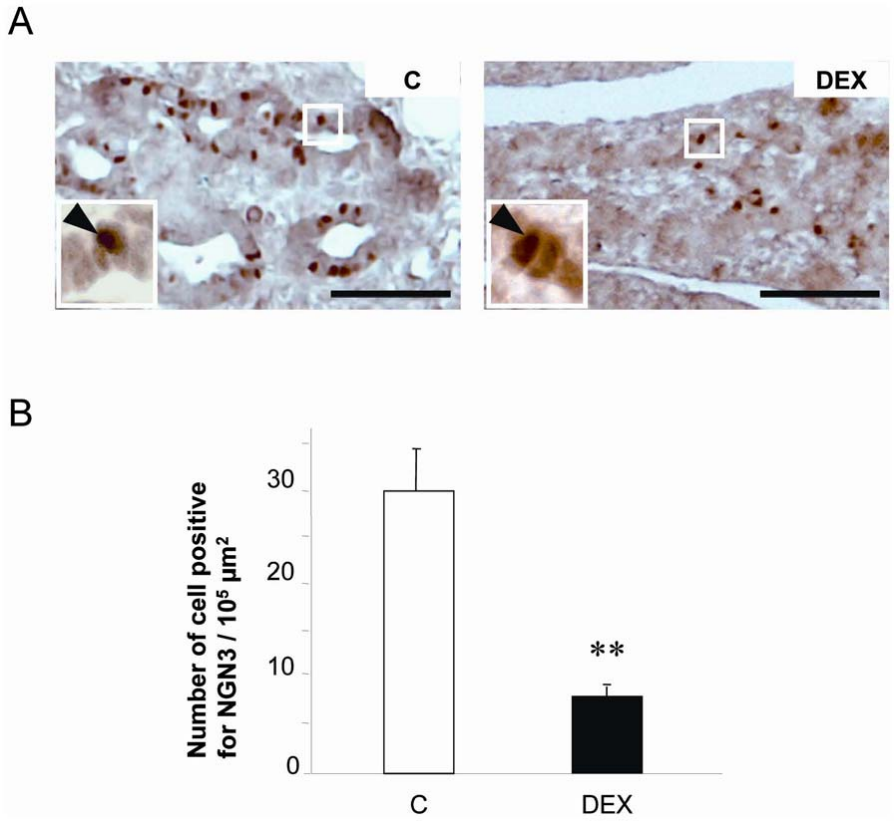
Effect of dexamethasone on NEUROG3 Expression in pancreas of 15-day-old fetuses. (A) Detection of NEUROG3 positive cells in the duct network at day 15 (brown; arrowheads in insets), n = 6, Scale bar = 25 µm. (B) The number of positive nuclei was evaluated per pancreas surface area (100 µm2). Values are means ± SEM, **P<0.01 vs Controls. Scale bar = 20 µm.

The number of cells immunoreactive for NEUROG3, a marker expressed in endocrine precursor cells, was measured on pancreatic sections at day E15 of gestation in DEX animals to assess the rate of cell commitment to the endocrine lineage (Figure 4A). Clearly, fetuses overexposed to GCs featured a 74% reduction in the number of NEUROG3-positive nuclei in the pancreatic epithelium (Figure 4B). To examine the differentiated status of the committed beta-cells in islets, the co-expression of insulin and PDX1 was also analyzed on pancreatic sections from 21-day old fetuses in the 3 groups. In all groups (C, DEX, DEXL), every beta-cells co-expressed insulin and PDX1 at this late fetal age which indicates a similar stage of maturation (*data not shown*). A normal immunostaining of PTF1A, the main transcription factor involved in the development of exocrine cells, was detected in all groups of fetuses at the same intensity (*data not shown*).

### Fetal islet vascularization

**Figure 5:**
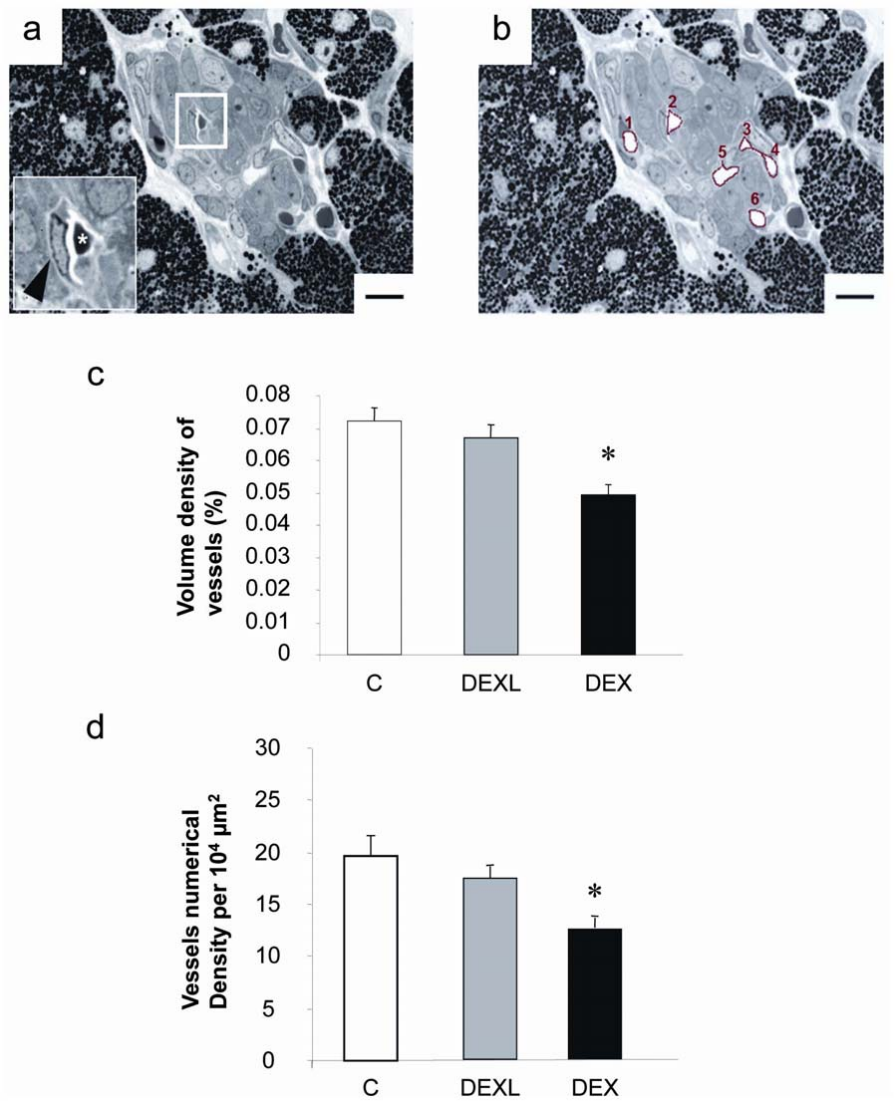
Effect of dexamethasone on islet vascularization of 21 day-old fetuses. (A and B) Semi-thin section (1 µm) of a pancreatic islet. Scale bar = 20 µm. (A) Inset: magnification of a blood vessel containing an erythrocyte (*). The arrow shows an endothelial cell. (B) The islet is surrounded by exocrine tissue and 6 blood vessels are highlighted in red in this islet. (C) Volumic and (D) numerical density of islet blood vessels as measured on semi-thin sections. Values are means ± SEM, n = 9,* P<0.05, vs Controls.

To study the consequences of GCs overexposure on fetal islet vascularization, the volume and number density of islet blood vessels was measured by morphometrical analysis on semi-thin sections in pancreas of 21 day-old rat fetuses (Figure 5A–5B). Both volume and number density were markedly decreased respectively by about 30 and 40% in islets of DEX group compared to Controls (Figure 5C–5D), whereas the administration of dexamethasone during only the last week of gestation did not affect the fetal islet vascularization.

### Effect of dexamethasone on plasma glucose, plasma insulin levels, and pancreatic insulin content

Dexamethasone treatment did not alter either the maternal or fetal plasma glucose level. It significantly reduced the fetal plasma insulin level (Table 3), which was decreased by 40% in the DEXL group and by 60% in the DEX group. DEX and DEXL fetuses had higher pancreatic insulin content (Table 3).

**Table 3:** Effect of dexamethasone on pancreatic insulin content (PIC, ng/mg), plasma glucose (mg/dL) and insulin level (ng/mL).

	C	DEXL	DEX
PIC (n = 12)	143±1	178±9*	171±7*
Fetal insulin (n = 20)	4.48±0.6	2.66±0.2**	1.75±0.15***
Fetal glucose (n = 20)	54.8±1.9	55.4±2.6	48.6±2.9
Maternal glucose (n = 4)	87.1±1.0	91.2±3.1	81±5.1

Values are means ± SEM,

***P<0.001,

**P<0.01,

*P<0.05, vs C.

### Insulin secretion by fetal islets in vitro

**Figure 6:**
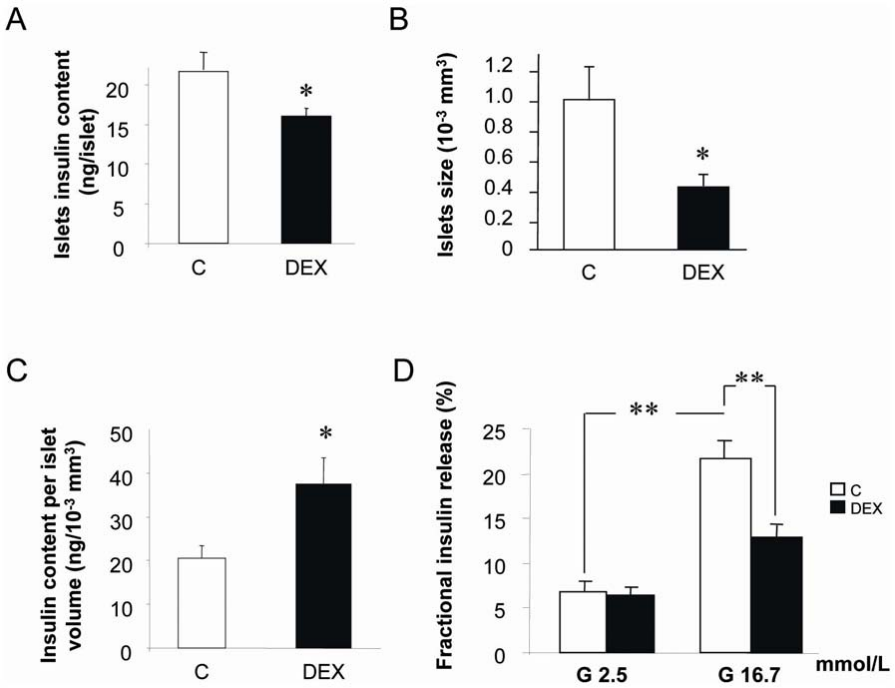
Effect of dexamethasone given to the mother throughout gestation on fetal neoformed islets. (A) Dexamethasone reduced the islet insulin content. Values are the means of 9 observations pooled from three independent cultures (n = 3). Bars represent SEM,* P<0.05 vs C. (B) Dexamethasone also reduced the size of islets after the culture ,* P<0.05 vs C. (C) The ratio islets insulin content per islet size was increased by dexamethasone treatment. Values are means of 100 observations pooled from 3 independent cultures. Bars represent SEM,* P<0.05. (D) Insulin secretion capacity of fetal islets. Islets were incubated in Krebs-Ringer medium containing glucose at 2.5 or 16.7 mmol/L. Values are the means of 9 observations pooled from three independent cultures (n = 3). SEM, ** P<0.01.

Batches of 10 fetal islets “neoformed” (see M&M) were incubated in Krebs-Ringer solution containing 2.5 mmol/L or 16.7 mmol/L of glucose. DEX islets collected after 7 days of culture were smaller and contained less insulin than Controls (Figure 6A–B). If the insulin content of neoformed islets was adjusted for their size, the insulin content per “islet volume” was increased in DEX group (Figure 6C) however they secreted less insulin in response to glucose stimulation. Indeed, although at low glucose concentration, both control and DEX islets released about 5% of their insulin content after 2 hours incubation, when control islets were stimulated with 16.7 mmol/L glucose, they released 22% of their insulin content, whereas DEX islets released only 12% which was not statistically different from the basal release (2.5 mmol/L) (Figure 6D).

## Discussion

This paper clearly shows that excessive glucocorticoid levels during gestation have dramatic consequences for the growth of the fetus and especially for the establishment of alpha and beta cell mass in the pancreas. When dexamethasone was given only during the last week of gestation, alpha and beta-cell mass was reduced due to impairment of cell commitment probably through a direct effect of dexamethasone. The novelty of our study is that when dexamethasone treatment was extended to the whole gestation period, fetal islet vascularization and beta-cell proliferation were additionally altered. This further contributes to amplify the reduction of the beta-cell mass resulting from the direct effect of dexamethasone later during fetal development. Taking together, our data suggest that the sensitive window of the endocrine pancreas to high GCs level is less narrow than previously suggested [13], [14]. In addition, we showed that beta cell function was altered after intrauterine exposure to high level of GCs.

Pancreas develops from the anterior midgut region of the endoderm epithelium through evaginations induced by adjacent mesodermal structures such as the notochord and the dorsal aorta [23], [24], [25]. The expression of Sonic hedgehog (*shh*) is suppressed whereas that one of *Pdx1* is stimulated. This occurs between E10.5 to E13.5 in mouse [26]. Later, the exo-endocrine specification is controlled by the Notch/Hes signaling system, which when active leads, in the exocrine and ductal progenitors and to the suppression of the pro-endocrine factor neurogenin-3, a transcription factor transiently expressed in pancreas, which controls the commitment of multipotent pancreatic endodermal progenitors to the endocrine fate [27], [28]. After the exo-endocrine specification, the endocrine cell differentiation is based on the successive expression of transcription factors which are different for each endocrine cell (for review see [23], [25], [29]. In addition to its early role in the pancreas development, *Pdx1* reappears later and intervenes in beta-cell differentiation.

In the mouse pancreas, the GR is only detected from E12 [30] and in rat, from E15 [14]. This suggests that the potential critical period in the pancreas for direct effect GCs susceptibility should be beyond E12 in mice and E15 in rat. However, the precise stage at which glucocorticoids act during pancreas development is still controversial. GCs have been shown to act during exo-endocrine specification favouring the exocrine differentiation at the expense of endocrine cells [13]. Indeed, when glucocorticoid was added *in vitro* to the culture medium, an increased expression of genes associated with exocrine development was observed while markers of endocrine differentiation were reduced [13]. On the other hand, Gesina and colleagues (2006) have shown that when GR null/null mice were studied at E15.5 they featured to be indistinguishable from wild-type regarding pancreatic size, tissue structure, beta cell fraction, Ngn3 and Pdx1 expression, as well as the production of transcription factors involved in exocrine differentiation [14]. These data suggest that the GCs control of beta cell development occurs after the Ngn3 production, thus after exo-endocrine specification. This relatively late effect of GCs on endocrine differentiation would suggest that the positive regulation of GCs on exocrine differentiation is secondary rather than part of an exo-endocrine switch mechanism. More recently, Valtat et al (2010) have shown that, in mice, the impact of maternal undernutrition on the impairment of beta cell expansion was mediated by the presence of glucocorticoid receptor (GR) on beta cell progenitors. The progeny of undernourished dams displayed a high level of GCs accompanied by a decrease number of precursor cells expressing *Ngn3* and *Pdx1* expression leading, to a reduction of beta- and alpha-cell mass. On the other hand, the expression of genes encoding for exocrine marker was increased in the pancreas of undernourished fetuses. When GCs-GR signaling was disrupted in underfed fetuses through Knocking-out GR in pancreatic precursor cells, the alteration observed in the wild type underfed were not observed in the KO [7]. These data indicate that GR signaling is important between progenitors and insulin-positive cells, but as mentioned by Valtat the precise stage at which this occurs is not yet clear.

Here we showed that at day 15, the pancreatic ducts from the DEX group expressed less Ngn3 positive cells. In consequence, the beta and alpha cell mass was lower in these animals on the last day of gestation, consistently with the hypothesis of GCs intervention in modulating the exocrine/endocrine balance. At day E21, the pancreas of offspring from mothers having received dexamethasone throughout gestation featured a normal organization. Indeed, the exocrine tissue exhibited a normal expression of PTF1A and the islets were composed of a core of beta-cells co-expressing PDX1 and insulin, surrounded by alpha- and delta-cells. When dexamethasone was given during the last week only, the pancreas of 21 day-old offspring displayed a normal organization but alpha and beta cell mass was less reduced compared to DEX pancreas. This suggests that the direct intervention of GCs on endocrine specification does not account for the complete reduction of endocrine mass observed in fetuses treated during the entire gestation period.

Proliferation is obviously involved in the modulation of the beta-cell mass. GCs have already been reported to reduce proliferation in various cell types [31], [32], [33], [34]. Despite extensive research effort, there is no consensus on the mechanisms by which GCs may reduce cell proliferation. In the present publication, we showed that at day 21 of pregnancy, beta cell proliferation was reduced when dexamethasone was given during the entire gestation period which led to less large islets and more small islets in this group. This observation is consistent with the recent finding of Valtat et al (2010). Indeed, when GR was lacking in pancreatic precursors cells, the proliferative index was increased in pancreatic beta cell [7]. In our experiment, when dexamethasone was given during the last week only, the proliferative index of beta cell was unchanged and the islet size distribution was similar to that of the controls. This suggests that the sensitive window of beta cell proliferation to GCs is placed before the onset of GR expression in rat pancreas.

Other tissues could contribute to the indirect effect of GCs on beta cell proliferation. GRs are expressed in the placenta and the liver earlier than in the pancreas, being already detected in these organs at day E9.5 in mice [15]. Both tissues are major producers of growth factors and are susceptible to GCs during development [35], [36], [37]. In our study, the growth of these organs was strongly affected by GCs over-exposure. Maternal glucocorticoid treatment appeared to reduce the placental transfer of glucose [38] and leptin [39], two master regulators of fetal growth. In another study, dexamethasone-induced IUGR was associated with dysregulated expression of IGF-II and prolactin in the junctional zone of the placenta [35]. It has been previously shown that dexamethasone was unable to reduce the proliferative activity of 6 day-old rat islets in vitro, except when the latter were stimulated by prolactin [40]. This supports the idea that anti-proliferative activity of GCs on beta-cell was mediated through indirect effect.

Islet vascular alteration may also be implicated in reduced beta-cell proliferation in DEX group. The influence of high GCs level on endothelial cells has already been reported in other tissues. Germinal matrix is a highly cellular and highly vascularized region in the brain from which cells migrate out during brain development. GC administration to pregnant rabbit reduced endothelial proliferation of this region in the developing brain of the progeny [41]. The same observation was made in germinal matrix of premature babies exposed to GC [41]. Then, it is possible that GC may have reduced the beta cell mass through an inhibitory effect on endothelial cell knowing the importance of the endothelium for islet development, highlighted during the last decade. Recently, signals from the endothelium that promote beta-cell proliferation were identified [16], [17], [42], [43], [44]. Here we showed that islet blood vessels were reduced in DEX group. In parallel the beta-cell proliferation was lower. This was not the case in DEXL group in which the first administration of the treatment took place at day F15, day at which islet vascularization can already be observed. Dexamethasone was probably administered too late to significantly influence the early mechanisms involved in islet angiogenesis. In this group, the beta-cell proliferation was also unaffected despite the beta-cell mass was lower. Interestingly, in ZDF rat, a model of type 2 diabetes, the beta-cell mass and function were impaired, together with an alteration of the islet vascular integrity [43]. It was proposed that such vascular alteration played a role in the beta-cell deficiency. In addition, in a recent study examining the process of beta-cell loss in IUGR rat offspring after birth, a reduced vascularity was observed in islet before the deterioration of beta cell mass [45]. These strong associations between islet vascularization and beta-cell proliferation reinforce the concept of a developmental association between endothelial cells and beta-cells.

To characterize the impact of dexamethasone on fetal beta cell function, we evaluated the insulin secretion in cultured neoformed islets from foetus of dexamethasone-treated mother. We showed that islet from DEX foetuses secreted less insulin in response to glucose compared to controls. These data are consistent with previous observations showing decreased glucose stimulated insulin secretion following glucocorticoid treatment *in vitro* [46]. The precise mechanisms mediating the inhibitory intervention of GCs on insulin secretion is still elusive [47], [48], [49], [50]. The programming of beta cell function was expected since a lasting alteration of the insulin secretion in adult offspring after dexamethasone treatment during early life has been recorded [51]. Indeed, prenatal exposure to dexamethasone produces fasting hyperglycaemia and/or glucose intolerance later in life.

In conclusion, the developing pancreas is sensitive to an excess of glucocorticoids *in vivo* which reduces the beta- and alpha-cell mass by different mechanisms according the stage of development during which they were applied. GCs may act directly on pancreas during the last week of gestation which corresponds to active endocrine neogenesis but when present from the beginning of the pancreas development, it reduces islet vascularization and pancreatic endocrine cell proliferation, which further compromises the beta and the alpha cell mass. So, our work shows new alterations of the endocrine pancreas development and proposes possible pathways to be investigated such as the contribution of the IGFs and islet vascularization. Because glucocorticoids are overproduced in stress condition that may be present throughout gestation and because they may program glucose intolerance in the progeny, this study provides further insights into the pathogenesis of common metabolic disorders.
